# Antitumor Effects of Ursolic Acid through Mediating the Inhibition of STAT3/PD-L1 Signaling in Non-Small Cell Lung Cancer Cells

**DOI:** 10.3390/biomedicines9030297

**Published:** 2021-03-13

**Authors:** Dong Young Kang, Nipin Sp, Jin-Moo Lee, Kyoung-Jin Jang

**Affiliations:** 1 Department of Pathology, School of Medicine, Institute of Biomedical Science and Technology, Konkuk University, Chungju 27478, Korea; kdy6459@kku.ac.kr (D.Y.K.); nipinsp@konkuk.ac.kr (N.S.); 2 Pharmacological Research Division, National Institute of Food and Drug Safety Evaluation, Osong Health Technology Administration Complex, Cheongju-si 28159, Korea; elzem@korea.kr

**Keywords:** ursolic acid, NSCLC, tumorsphere, EGFR, STAT3, MMP2, PD-L1

## Abstract

Targeted therapy based on natural compounds is one of the best approaches against non-small cell lung cancer. Ursolic acid (UA), a pentacyclic triterpenoid derived from medicinal herbs, has anticancer activity. Studies on the molecular mechanism underlying UA’s anticancer activity are ongoing. Here, we demonstrated UA’s anticancer activity and the underlying signaling mechanisms. We used Western blotting and real-time quantitative polymerase chain reaction for molecular signaling analysis. We also used in vitro angiogenesis, wound healing, and invasion assays to study UA’s anticancer activity. In addition, we used tumorsphere formation and chromatin immunoprecipitation assays for binding studies. The results showed that UA inhibited the proliferation of A549 and H460 cells in a concentration-dependent manner. UA exerted anticancer effects by inducing G0/G1 cell cycle arrest and apoptosis. It also inhibited tumor angiogenesis, migration, invasion, and tumorsphere formation. The molecular mechanism underlying UA activity involves UA’s binding to epidermal growth factor receptor (EGFR), reducing the level of phospho-EGFR, and thus inhibiting the downstream JAK2/STAT3 pathway. Furthermore, UA reduced the expressions of vascular endothelial growth factor (VEGF), metalloproteinases (MMPs) and programmed death ligand-1 (PD-L1), as well as the formation of STAT3/MMP2 and STAT3/PD-L1 complexes. Altogether, UA exhibits anticancer activities by inhibiting MMP2 and PD-L1 expression through EGFR/JAK2/STAT3 signaling.

## 1. Introduction

Non-small cell lung cancer (NSCLC), one of the common cancers, accounts for approximately 85% of all lung cancer incidence, 70% of which may lead to metastasis, resulting in death. Various factors can induce tumorigenesis in NSCLC; tobacco is considered the primary factor [1]. There are some standard treatment methods for cancer. Targeted therapy is an effective method, as the alteration of molecular signaling pathways is a well-known factor of NSCLC tumorigenesis. Aberrations in major signaling pathways, such as epidermal growth factor receptor (EGFR), tyrosine kinases, and molecular pathways that control cancer hallmarks such as angiogenesis, cell cycle, apoptosis, and proteosome regulation, finally result in metastasis. EGFR overexpression is associated with approximately 80% of NSCLC incidence with a poor prognosis rate [2]. Several commercially available inhibitors that target EGFR are used to treat lung cancer, but they cause some side effects and lack survival benefits [3]. Vascular endothelial growth factor (VEGF) is a ligand that binds to VEGF receptors, especially VEGF-R2, which is necessary for tumor progression processes such as vascular permeability and angiogenesis. VEGF is also considered an impactful target in lung cancer treatment [4].

The major checkpoints are uncontrolled in tumorigenesis, leading to cell cycle progression, the inability to induce apoptosis, and the suppression of antitumor molecules or tumor suppressor genes. Aberrations in cell division or cell cycle process could induce uncontrolled cell division, causing tumorigenesis. Cyclin-dependent kinases (CDKs) are critical regulators of the cell cycle; CDK inhibitors play a suppressive role and prevent normal cells from becoming cancerous. A CDK inhibitor, p21 (p21^WAF1/Cip1^), arrests the cell cycle when DNA damages occur [5]. It acts as a tumor suppressor in response to stimuli. It can also inhibit tumor suppressor protein 53 (p53)-independent cell proliferation; as such, p21 is also known as a master effector of the tumor suppressor pathways [6]. The proteins p21 and p27 (KIP1) can inhibit CDK proteins as well as cyclin proteins such as cyclin D1; therefore, their anticancer activity depends on the enhancement of p21 or p27 and the down-regulation of cyclin and CDK proteins [7]. Cell cycle arrest leads to apoptosis and DNA damage response mechanisms. When a normal cell cannot induce apoptosis or DNA repair mechanisms by stimuli such as mutation, this condition promotes tumorigenesis. The loss of the ability to induce apoptosis is a critical factor in tumor development, and the method could also be used for anticancer treatments [8,9].

Promoting angiogenesis is considered one of the hallmarks of cancer [10,11]. Cancer cell growth, differentiation, and tumor metastasis mainly depend on angiogenesis triggered by stimuli [12]. Tumor cells need vascular support for the blood flow to supply enough oxygen for cell growth; the lack of vascular support may even lead to apoptosis [13]. During angiogenesis, multiple angiogenic activators such as VEGF, basic fibroblast growth factor (bFGF), angiogenin, transforming growth factor (TGF)-α, TGF-β, and tumor necrosis factor (TNF)-α become activated to promote the molecular signaling of tumor angiogenesis [14]. VEGF activation also promotes the activation of metalloproteinases (MMPs), which destroy the extracellular matrix and allow endothelial cells to migrate and invade, resulting in tumor metastasis [15]. Thus, VEGF and MMP proteins are considered critical targets in cancer treatment [16].

Cancer stem cells (CSCs) cause cancer recurrence and promote tumorigenesis. Gene mutations or stimuli can activate CSCs by activating angiogenesis, suppressing cell cycle arrest, inducing apoptosis, and finally resulting in metastasis [17]. Traditional cancer therapies reduce tumor size, but CSCs are resistant to conventional therapies. Targeted therapy against CSCs could eliminate the risk of cancer recurrence [18]. Tumorsphere formation has been widely used to evaluate the stemness properties of cancer cells, whereby differentiated cells undergo cell death, and CSCs survive and proliferate to form tumorspheres [19]. Tumorsphere formation is considered a treatment platform for CSCs in NSCLC cells. Lung tumorspheres are highly resistant to many chemotherapeutic agents. This idea could be useful for developing new drugs against NSCLC [20,21]. The tumorspheres from cancer cells give support to the independence of external stimuli. This can provide an experimental system for identifying the factors that play vital roles in the molecular signaling of CSCs during the screening of a new drug against NSCLC [22].

*EGFR* mutation or overexpression is often observed in NSCLC cells. It signals toward its downstream targets, which then translocate into the nucleus to promote transcription and tumor progression. Janus kinase 2 (JAK2) and signal transducer and activator of transcription 3 (STAT3) signaling is an essential pathway in human cancers, as well as CSCs, acting by regulating inflammatory cytokines such as interleukin (IL)-6 [23]. The JAK2/STAT3 pathway participates in cancer cell survival, proliferation and progression by regulating multiple processes, such as epithelial–mesenchymal transition (EMT), which is required for tumor metastasis, and molecular signals that control other cancer hallmarks [24]. The programmed death ligand-1 (PD-L1)/programmed cell death protein 1 (PD-1) pathway is a vital checkpoint for tumor-induced immune escape that is mediated through T-cell exhaustion. In NSCLC, PD-L1 (CD274) is found to be overexpressed and regulated through EGFR/JAK/STAT3 signaling [25,26]. Some studies showed that high PD-L1 expression was associated with tumor metastasis, cancer recurrence, and tumor invasion; PD-L1 could be considered an independent element in evaluating immunotherapy during metastasis [27,28]. As such, PD-L1 could play a crucial role in the immune microenvironment between the primary tumor and the secondary metastatic tumor; PD-L1 can help increase the understanding of cancer’s response to immunotherapy and develop PD-L-targeted therapy [29].

Targeted anticancer therapy using natural compounds is an effective approach because the natural compounds are efficacious and have fewer adverse effects. Ursolic acid (UA) is a pentacyclic triterpenoid derived from fruits and medicinal herbs with pharmaceutical and biological effects [30]. It can act against various cancer-related processes, such as the induction of apoptosis, the suppression of inflammatory responses, tumor metastasis, angiogenesis, and antioxidation. On the other hand, UA derivatives are also found to have pharmacological applications related to disease prevention [31]. The molecular signaling of UA is primarily linked to pro-inflammatory cytokines such as IL-7, IL-17, IL-1β, TNF-α or cyclooxygenase-2, and nitric oxide synthase through nuclear factor-κB, the primary factor in inflammatory responses to external stimuli [32]. In breast cancer and gastric cancer cells, UA induces cell cycle arrest and inhibits cell proliferation by inducing intrinsic and extrinsic pathways of apoptosis in vitro as well as in vivo [33,34]. UA can also induce cancer cell death and reduced tumor growth by regulating the autophagy-related gene 5-dependent autophagy in cervical cancer cells [35]. In NSCLC, UA has been found to have anticancer effects through the inhibition of autophagy and the suppression of TGF-β1-induced EMT, via regulating integrin αVβ5/MMPs signaling [36,37]. However, the role of UA signaling in the inhibition of PD-L1 in NSCLC remains to be elucidated.

In this study, we aim to determine UA’s anticancer effects on processes such as cell cycle arrest, apoptosis, angiogenesis, migration, invasion, and tumorsphere formation in NSCLC cells. We also aimed to investigate PD-L1’s role in UA-mediated anticancer activities and the underlying molecular mechanisms.

## 2. Materials and Methods

### 2.1. Antibodies and Cell Culture Reagents

Roswell Park Memorial Institute-1640 (RPMI-1640) medium, penicillin–streptomycin solution, and trypsin-EDTA (0.05%) (Gibco, Thermo Fisher Scientific, Inc., Waltham, MA, USA) were purchased. UA (U6753) and fetal bovine serum (FBS) (Sigma-Aldrich, Merck KGaA, St. Louis, MO, USA) were obtained. The primary antibodies against CDK4 (sc-260), cyclin E (sc-481), VEGF (sc-507), MMP9 (sc-13520), and β-actin (sc-47778) with anti-mouse (sc-516102) and anti-rabbit (sc-2357) secondary antibodies (Santa Cruz Biotechnology, Dallas, TX, USA) were procured. The antibodies against p21 (#2974), p27 (#3686), pEGFR (#3777), EGFR (#4267), pJAK2 (#3776), JAK2 (#3230), pSTAT3 (#9145), and STAT3 (#9139) (Cell Signaling Technology, Beverly, MA, USA) were obtained. The antibodies against SOX2 (#MAB4423), OCT4 (#MABD76), NANOG (#MABD24), and MMP3 (#AB2963) were supplied by Merck Millipore (Burlington, MA, USA). The Cyclin D1 (ab6152) antibody (Abcam, Cambridge, MA, USA), MMP2 (E90317) antibody (EnoGene, New York, NY, USA), and the PD-L1 (R30949) antibody (NSJ Bioreagents, San Diego, CA, USA) were procured.

### 2.2. Cell Culture and Treatment

A549 (no. 10185) and H460 (no. 30177, Korean Cell Line Bank, Seoul, South Korea) cell lines were cultured in RPMI-1640 supplemented with 10% FBS and 1% penicillin–streptomycin at 37 °C and 5% CO_2_. The cells were grown to 80% confluency, gently washed twice with phosphate-buffered saline (PBS), and treated with various concentrations of UA at 37 °C for different durations according to the experimental design.

### 2.3. Cell Proliferation Inhibition

Cell viability was measured using the 3-(4,5-Dimethylthiazol-2-yl)-2,5-Diphenyltetrazolium Bromide (MTT) assay. Briefly, the cells were resuspended in RPMI-1640 and seeded in 96-well culture plates at a density of 3 × 10^3^ cells per well 1 day before drug treatment. During drug treatment, the cells were incubated with increasing concentrations of UA from 1 to 100 µM for 24 h. The vehicle control was incubated with a fresh medium containing dimethyl sulfoxide (DMSO). Following drug treatment, MTT at 5 mg/mL was added to the cells to incubate for 4 h at 37 °C. The resulting formazan crystals were dissolved in DMSO and read at the absorbance of 560 nm on an ultra-multifunctional microplate reader (Tecan, Durham, NC, USA). All measurements were performed in triplicates, and the experiments were repeated at least three times.

### 2.4. DAPI Staining and Morphological Analysis

Chromatin condensation during apoptosis was examined with DAPI staining solution (ab228549, Abcam, Cambridge, MA, USA). A549 and H460 cells were seeded in 6-well plates at a density of 1.5 × 10^5^ cells/well, treated with 10 or 20 µM UA for 24 h, and washed twice with PBS. They were then fixed with 200 µL of 100% methanol for 10 min, and washed twice with PBS. Then, 500 µL of 300 nM DAPI staining solution was added. The cells were washed twice with PBS, mounted with the mounting solution on microscope slides, and observed with fluorescence microscopy (Olympus IX71/DP72, Tokyo, Japan).

### 2.5. Western Blotting

Whole-cell lysates were prepared by incubating the untreated or UA-treated A549 and H460 cells on ice with the RIPA lysis buffer (20–188; EMD Millipore) containing protease and phosphatase inhibitors. Protein concentrations were measured using the Bradford assay (Thermo Fisher Scientific, Inc., Waltham, MA, USA). Equal amounts of protein at 100 µg/well were resolved with 10–15% sodium dodecyl sulphate–polyacrylamide gel electrophoresis (SDS-PAGE). The separated proteins were then transferred onto nitrocellulose membranes. The blots were blocked for 1 h with 5% skim milk (BD Biosciences, San Jose, CA, USA) in TBS-T buffer (20 mM Tris–HCl) (Sigma-Aldrich; Merck KGaA, St. Louis, MO, USA), pH 7.6, 137 mM NaCl (Formedium, Norfolk, UK; NaCO_3_), and 0.1 × Tween 20 (Scientific Sales, Inc., Oak Ridge, TN, USA). Next, the membranes were incubated with primary antibodies (0.5–1 µg/mL) diluted in 5% bovine serum albumin (EMD Millipore) overnight at 4 °C on a shaker, washed with TBS-T, incubated with HRP-conjugated secondary antibodies (1–2 µg/mL) for 1 h at room temperature, and detected using a Femto clean enhanced chemiluminescence solution kit (GenDEPOT; 77449; Katy, TX, USA) and an LAS-4000 imaging device (Fujifilm, Tokyo, Japan).

### 2.6. Reverse Transcriptase–Quantitative Polymerase Chain Reaction (RT-qPCR)

Total RNA was extracted from the cells using the RNeasy Mini Kit (Qiagen GmbH, Hilden, Germany). The extracted RNA was quantified with a spectrophotometer (NanoDrop™ 1000, Thermo Fisher Scientific, Inc., Waltham, MA, USA) at 230 nm and reverse transcribed into cDNA at 42 °C for 1 h and 95 °C for 5 min using a first-strand cDNA synthesis kit (Bioneer Corporation, Daejeon, South Korea) and oligo d(T) primers. The RT-PCR premix kit (Bioneer Corporation, Daejeon, Korea) was used to amplify *CCND1*, *CCNE1*, *CDK4*, *CDKN1A*, *CDKN1B*, *MMP2*, *MMP3*, *MMP9*, *VEGF*, *CD274*, *SOX2*, OCT4 (*POU5F1)*, *NANOG* and *GAPDH* cDNA with the corresponding primers (Bioneer Corporation) (Table S1). The qPCR mix consisted of 2 µL of diluted cDNA, 1 µL each of the forward and reverse primers at 100 pM, and 10 µL of the TB Green Advantage Premix (Takara Bio, Shiga, Japan). The qPCR process included initial denaturation at 95 °C for 5 min, followed by 40 cycles of denaturation at 95 °C for 40 s, annealing at 58 °C for 40 s, extension at 72 °C for 40 s, and a final extension at 72 °C for 5 min in a thermal cycler (C1000 Thermal Cycler, Bio-Rad, Hercules, CA, USA). The relative expression of the target genes was normalized to the expression of *GAPDH* and calculated using the Cp values. All the measurements were performed in triplicates.

### 2.7. Cell Cycle Analysis

The DNA content of the UA-treated and non-treated cells was determined using a BD Cycletest Plus DNA Reagent Kit (BD Biosciences, CA, USA). Approximately 5 × 10^5^ cells were treated with or without UA for 24 h, washed with PBS, and permeabilized with trypsin. The RNA interaction with propidium iodide (PI) was neutralized by treating the cells with RNase and trypsin inhibitor. The samples were then stained with PI for 30 min in the dark at room temperature and analyzed using a FACSCalibur flow cytometer (BD Biosciences, San Jose, CA, USA).

### 2.8. Apoptosis Analysis

Annexin V-FITC (BD556547, BD Pharmingen, CA, USA) was used to measure the level of apoptosis in A549 and H460 cells. UA-treated cells were washed with PBS, resuspended in the binding buffer to a concentration of 1 × 10^6^ cells, and stained with annexin V-FITC and PI for 10 min in the dark at room temperature. The percentage of apoptotic cells was measured by flow cytometry using the FACSCalibur (BD) and analyzed using the FlowJo software (v10).

### 2.9. In Vitro Angiogenesis Assay

ECMatrix (ECM625, Merck KGaA, 64293 Darmstadt, Germany) was thawed at 4 °C overnight. Then, pre-chilled 96-well plates were incubated with 50 µL diluted ECMatrix at 37 °C for 1 h for ECMatrix to solidify. Next, 150 µL of human vascular endothelial cells (HUVECs) at 1 × 10^4^ with or without UA were added to the solidified matrix and incubated at 37 °C for 12 h. Afterward, endothelial cell formation was assayed with the in vitro angiogenesis kit (Millipore, Billerica, MA, USA) using a microscope. The focus was placed on distinct areas, and the tubes that formed were counted according to the kit’s instructions.

### 2.10. Matrigel Invasion Assay

The invasion assay was conducted with ready-to-use Transwell invasion chambers pre-coated with Matrigel (BD Biocoat, MA, USA). First, 5 × 10^4^ cells were added to each insert. The drug-containing media were added to the receiver plate, and the inserts containing cells were placed onto it. After a 24 h incubation in a humidified chamber at 37 °C, the cells on the upper surface were removed using a cotton swab, and the cells that had invaded the inserts’ apical surface were stained with crystal violet and observed under a microscope. The focus was placed on four distinct areas, and the cells were counted.

### 2.11. Wound Healing Assay

A549 and H460 cells were seeded in 6-well plates at 1 × 10^5^ cells/well in RPMI-1640 media and incubated for 24 h. After the cells became confluent, the cell layers were scratched with a pipette tip, washed with PBS to remove the debris, and treated with UA for 24 h. The control cells were not treated. The wound edges were photographed at different time intervals under a microscope to measure the relative area of wound closure using ImageJ software (NIH Image, Bethesda, MD, USA).

### 2.12. Tumorsphere Formation Assay

The A549 and H460 cells were cultured with or without UA and STAT3 siRNA in DMEM/F12 media containing growth supplements, EGF, bFGF, and B27, in low-attachment 6-well plates. The treatment day was considered day 0, and the incubation with UA continued for 14 days. Photographs were taken on days 0, 7, and 14 under a microscope. Total RNA and protein were isolated from the tumorsphere and analyzed using real-time qPCR and western blotting, respectively.

### 2.13. siRNA Transfection

The A549 and H460 cells were seeded in 6-well plates at 1 × 10^6^ cells per well, grown to 60% confluence, and transfected with STAT3 siRNA (sc-29493; Santa Cruz Biotechnology, Dallas, TX, USA) using the lipofectamine transfection reagent (Thermo Fisher Scientific, Inc., Waltham, MA, USA). After 24 h, the transfected cells were then cultured with or without UA for another 24 h under the same cell culture conditions.

### 2.14. Molecular Docking

Molecular docking was used to identify UA’s binding site in the EGF receptor (EGFR) using PyRx software (v0.99) in the AuotDock Vina platform (Scripps Research Institute, San Diego, CA, USA). UA’s 3D structure was obtained from PubChem (ID: 64945) and EGFR’s 3D structure from Protein Data Bank (PDB ID: 2GS2). The obtained docked products were visualized using PyMol software (0.8).

### 2.15. Chromatin Immunoprecipitation (ChIP) Assay

A ChIP assay was performed using an imprint chromatin immunoprecipitation kit according to the manufacturer’s protocol. The A549 and H460 cells were fixed with 1% formaldehyde, quenched with 1.25 M glycine, resuspended in the nuclei preparation buffer, and sonicated in shearing buffer under optimized conditions. The sheared DNA was diluted in dilution buffer at the ratio of 1:1. The diluted supernatant was then incubated with the antibody against STAT3 in pre-coated wells for 90 min. Normal mouse IgG and anti-RNA polymerase II were used for negative and positive controls, respectively. The unbound DNA was washed off with the IP wash buffer, whereas the bound DNA was collected by cross-link reversal using a DNA release buffer containing proteinase K. The released DNAs and the DNA from the internal controls were purified using the GenElute Binding Column G and quantified using specific MMP2 and PD-L1 primers (Table S1) by real-time qPCR.

### 2.16. Statistical Analyses

All the experiments were performed at least three times. The results are expressed as the mean ± standard error of the mean. Statistical analyses were conducted using one-way analysis of variance (ANOVA) or Student’s *t*-test. One-way ANOVA was performed with Tukey’s test for post hoc analysis. The analyses were performed using the SAS 9.3 software program (SAS Institute, Inc., Cary, NC, USA). A difference with a *p*-value <0.05 (*) was considered statistically significant.

## 3. Results

### 3.1. UA Inhibits NSCLC Cell Proliferation in a Concentration-Dependent Manner

**Figure 1:**
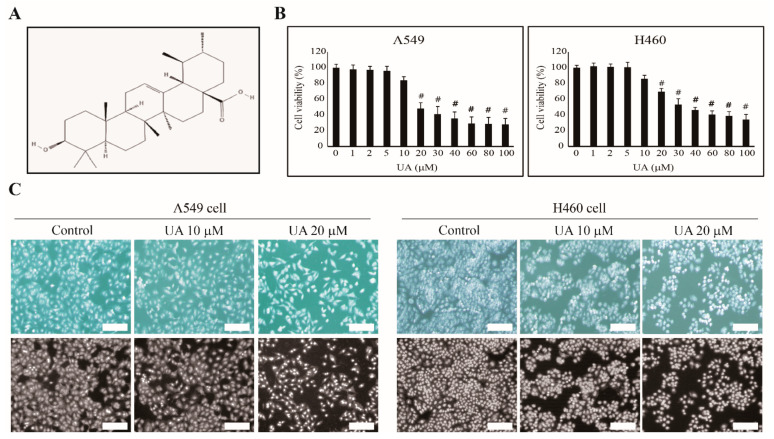
Ursolic acid (UA) inhibited non-small cell lung cancer (NSCLC) cell proliferation. (**A**) The structure of UA. (**B**) The MTT assay showed that the cell proliferation of A549 and H460 was inhibited by increasing UA concentrations for 24 h. (**C**) UA induced nuclear aberrations in NSCLC cells. Phase-contrast microscopy images showing abnormal nucleus formation induced by 10 and 20 µM UA in A549 and H460 cells. Representative photographs are presented (scale bar: 100 µm). The values of three independent experiments performed in triplicates (*n* = 3) were represented as mean ± SEM. The controls were set to 100. # *p* < 0.001 vs. control.

First, we hypothesized that UA (structure in Figure 1A) could induce the apoptosis of NSCLC cells, A549 and H460. We examined UA’s inhibition of A549 and H460 cell proliferation at increasing concentrations from 1 to 100 µM for 24 h. We observed a concentration-dependent inhibition of cell proliferation starting at 20 µM UA on A549 and H460 cells (Figure 1B). Approximately 50% of the A549 and H460 cells died when treated with 20 and 30 µM UA, respectively. Thus, we used 10 and 20 µM UA for our further studies to show UA’s concentration-dependent effects. We also evaluated the apoptosis of the A549 and H460 cells at 10 and 20 µM UA, respectively, using morphological analysis with DAPI staining (Figure 1C). We observed a reduction in cell number at increasing concentrations of UA, confirming the anticancer activity of the natural compound, UA.

### 3.2. UA Induces Cell Cycle Arrest and Apoptosis in NSCLC Cells

**Figure 2:**
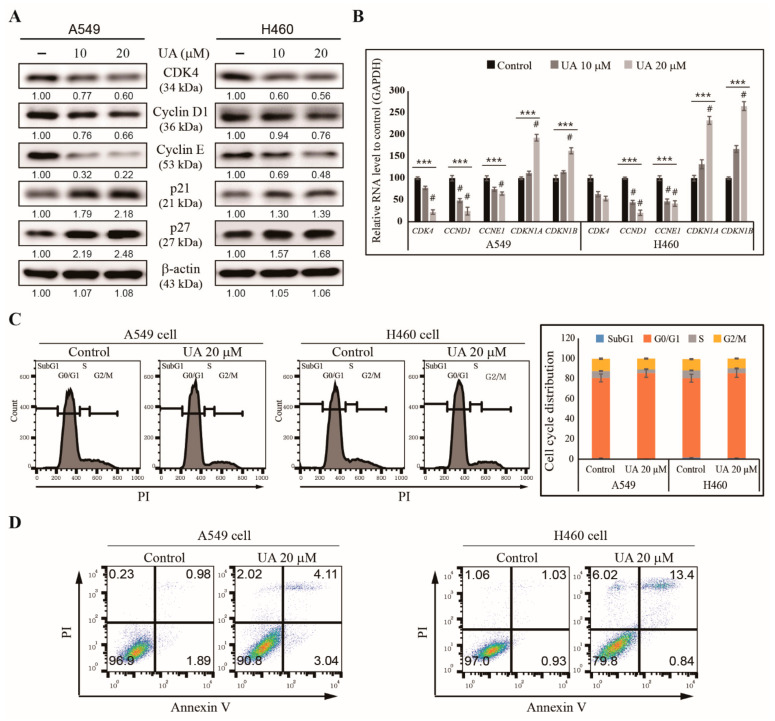
UA induced cell cycle arrest and apoptosis in NSCLC cells. (**A**) The western blotting for CDK4, cyclin D1, cyclin E1, p21 (CDKN1A), and p27 (CDKN1B) in A549 and H460 cells treated with 10 and 20 µM UA, respectively, for 24 h. β-actin was used as the housekeeping gene. (**B**) RT-qPCR analysis of the expression of cell cycle checkpoint genes *CCND1*, *CCNE1*, *CDK4*, *CDKN1A*, and *CDKN1B* in A549 and H460 cells after treatment with 10 and 20 µM UA, respectively, for 24 h. The Cp values were normalized to *GAPDH* mRNA. Controls are set to 100. (**C**) Flow cytometry analysis with PI staining of the cell cycle distribution in A549 and H460 cells after treatment with 20 µM UA for 24 h, with a graphical representation of G0/G1 arrest by UA in NSCLC cells. (**D**) Flow cytometry analysis using annexin V and PI staining in A549 and H460 cells after treatment with 20 µM UA for 24 h. *** *p* < 0.001 (ANOVA test), and # *p* < 0.001 vs. control.

We then examined the mechanisms underlying the inhibition of NSCLC cell proliferation by UA. We hypothesized that UA could induce cell cycle arrest and apoptosis. First, we analyzed the cell cycle checkpoint proteins in the UA-treated A549 and H460 cells using western blotting, and found reduced levels of CDK4, cyclin D1 and cyclin E, and increased levels of tumor suppressor proteins p21 and p27, with UA treatment (Figure 2A). We confirmed these patterns in the mRNA levels of the genes by observing that UA significantly inhibited the expressions of *CDK4*, *CCND1* and *CCNE1*, and upregulated the expressions of *CDKN1A* and *CDKN1B* in the NSCLC cells (Figure 2B). These results suggested UA’s ability to induce cell cycle arrest in NSCLC cells. Then, we analyzed the cell cycle distribution of the UA-treated A549 and H460 cells using flow cytometry. We found an arrest in the G0/G1 phase in the cell cycle caused by UA treatment (Figure 2C), suggesting UA’s ability to induce apoptosis. Therefore, we analyzed apoptosis induction by UA in NSCLC cells using flow cytometry, and observed an increased number of dead cells caused by 20 µM UA in both the A549 and H460 cells (Figure 2D). These results suggested that UA could be a candidate drug against NSCLC.

### 3.3. UA Inhibits Angiogenesis, Invasion, and Migration of NSCLC Cells

**Figure 3:**
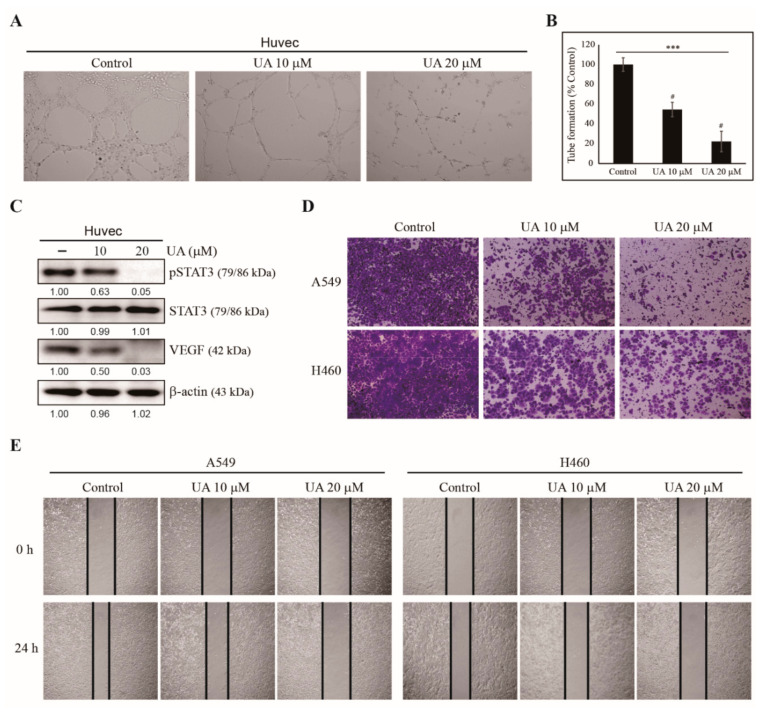
UA inhibited NSCLC cell angiogenesis, invasion, and migration. (**A**) The inhibition of angiogenesis by 10 and 20 µM UA during the in vitro angiogenesis assay of A549 and H460 cells, respectively. (**B**) Graphical representation of the in vitro angiogenesis assay showing the relative inhibition of tube formation. (**C**) Western blotting of A549 and H460 cells treated with 10 and 20 µM UA, respectively, for 24 h for phospho-STAT3, STAT3, and VEGF. The relative levels of the proteins were determined by densitometry and normalized to that of β-actin, which was set to 100. The data were confirmed by repeating the experiment three times. (**D**) The Matrigel invasion assay showing the inhibition of invasion in A549 and H460 cells treated with 10 and 20 µM UA, respectively, for 24 h. Scale bars: 100 µm. (**E**) The wound healing assay demonstrated the inhibition of migration in A549 and H460 cells treated with 10 and 20 µM UA, respectively, for 0 and 24 h. Scale bars: 50 µm. Statistical analysis was conducted using ANOVA test; *** *p* < 0.001; # *p* < 0.001 vs. control.

We have observed the induction of cell cycle arrest and apoptosis by UA in A549 and H460 NSCLC cells. Because UA plays a role against other cancer processes, such as tumor angiogenesis, migration, and invasion, we evaluated UA’s ability to suppress angiogenesis. HUVEC were used for in vitro angiogenesis assay with an extracellular matrix solution (Figure 3A). Angiogenesis was measured via tube formation with HUVECs in an extracellular matrix gel. The treatments of 10 and 20 µM of UA significantly reduced the tube formations in A549 and H460 cells, respectively (Figure 3B), indicating UA’s inhibition of angiogenesis. We examined UA for the inhibition of angiogenesis, and analyzed the proteins derived from HUVECs treated with or without UA with western blotting. UA-treated cells showed decreased VEGF levels and phosphorylated STAT3, which played a crucial role in angiogenesis; the total amount of STAT3 remained the same for the control and UA treatments (Figure 3C). These results suggest that UA can inhibit angiogenesis. Then, we studied whether UA could suppress tumor metastasis using an invasion assay with Matrigel (Figure 3D) and a wound healing migration assay (Figure 3E). We found decreased cell invasion and wound closure with increasing concentrations of UA in both A549 and H460 cells. These results suggest the antimetastatic activity of UA against NSCLC cells.

### 3.4. UA Suppresses Cancer Stemness by Inhibiting Tumorsphere Formation in NSCLC Cells

**Figure 4:**
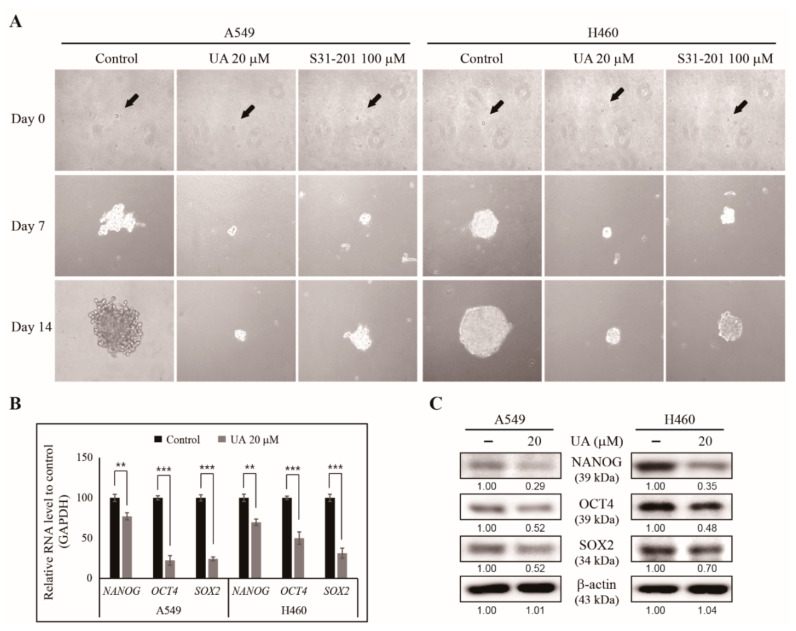
UA inhibited tumorsphere formation from NSCLC cells. (**A**) Tumorsphere formation was inhibited by 20 µM UA or 100 µM S3I-201 treatment after the A549 and H460 cells were cultured in DMEM/F12 media containing epidermal growth factor (EGF), basic fibroblast growth factor (bFGF) and B27 for 14 days. Photographs were taken on days 0, 7, and 14. Black arrows indicate the single cells on day 0. (**B**) RT-qPCR of the mRNA isolated from the A549 and H460 tumorspheres to demonstrate the expression of CSC marker genes after treatment with 20 µM UA for 24 h. The representative expressions of *NANOG*, *OCT4* and *SOX2* are shown. The Cp values were normalized to the level of *GAPDH* mRNA, which was set to 100. (**C**) Western blotting of the CSC marker proteins, NANOG, OCT4, and SOX2, in A549 and H460 tumorspheres after treatment with 20 µM UA for 24 h. The relative levels of proteins were determined by densitometry and normalized to that of β-actin, which was set to 100. The data were confirmed after repeating the experiment 3 times. ** *p* < 0.01 and *** *p* < 0.001 (Student’s *t*-test).

We have found that UA can inhibit the hallmarks of cancer in NSCLC cells. However, its ability to inhibit CSCs is unknown. Tumorspheres can only form from CSCs. Thus, we conducted a tumorsphere formation assay to analyze UA’s ability to inhibit cancer stemness in NSCLC cells. We cultured A549 and H460 cells in tumorsphere media in the presence of UA and 100 µM S3I-201, a STAT3 inhibitor, for 14 days, and photographed them under a microscope. After 14 days, the UA- and S31-201-treated cells showed a significant reduction in tumorsphere compared with untreated A549 and H460 cells (Figure 4A). These data suggest that UA can inhibit CSCs through STAT3 signaling. We confirmed CSC formation by analyzing the expression levels of CSC markers, *NANOG*, *POU5F1* and *SOX2*, in the UA-treated NSCLC cells, and found these genes to be significantly inhibited (Figure 4B). The inhibition of the CSC markers by UA was confirmed by probing the levels of NANOG, OCT4 and SOX2 in the tumorspheres using western blotting (Figure 4C). These results suggest UA’s ability to target CSCs.

### 3.5. UA Inhibits PD-L1 Expression Through the EGFR/JAK2/STAT3 Pathway in NSCLC Cells

**Figure 5:**
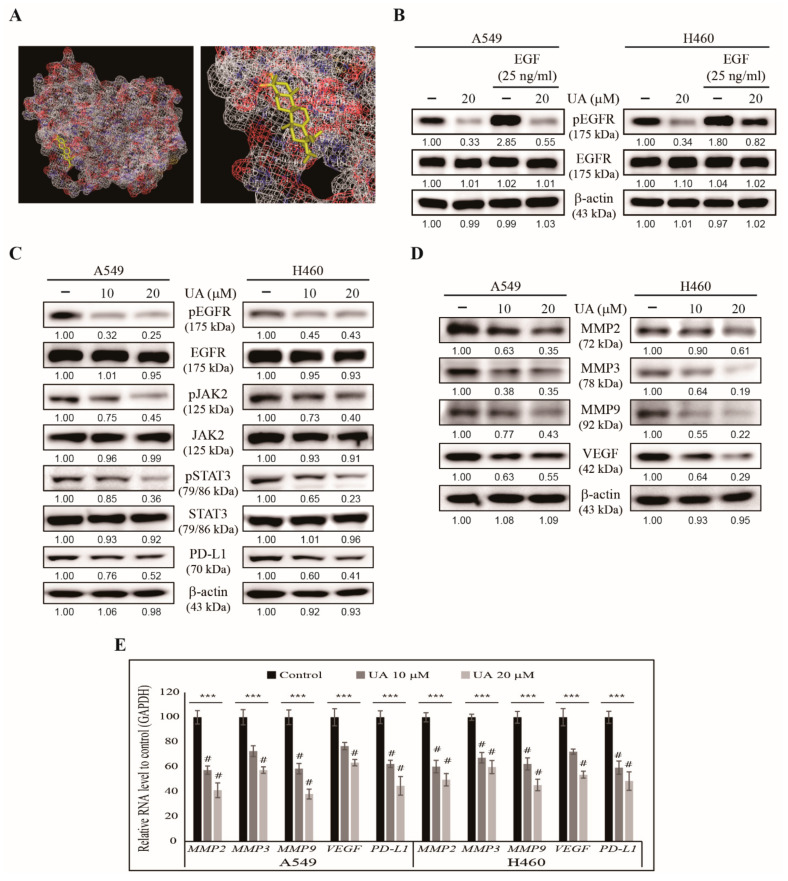
UA regulated EGFR/JAK2/STAT3 signaling in NSCLC cells. (**A**) Molecular docking using AutoDock Vina software showed UA binding (PubChem ID: 64945) to the ATP-binding domain of EGFR (PDB ID: 2GS2). (**B**) Western blotting of the lysate of the A549 and H460 cells pre-treated with recombinant EGF (25 ng/mL) for 1 h and then with 20 µM UA for 24 h, showing the levels of phosphorylated EGFR and total EGFR. The representative levels of the proteins were determined by densitometry and normalized to β-actin, which was set to 100. The data were confirmed after repeating the experiment 3 times. (**C**) Western blotting of the proteins involved in EGFR/JAK2/STAT3 signaling and PD-L1 in the A549 and H460 cells treated with 10 and 20 µM UA, respectively, for 24 h. β-Actin was used as the housekeeping protein. (**D**) Western blotting for MMPs and VEGF in the A549 and H460 cells treated with 10 and 20 µM UA, respectively, for 24 h. β-actin used as a housekeeping protein. **(E)** RT-qPCR analysis for the expression of *MMP2*, *MMP3*, *MMP9*, *VEGF*, and *PD-L1* in the A549 and H460 cells treated with 10 and 20 µM UA, respectively, for 24 h. The representative expressions of *MMP2*, *MMP3*, *MMP9*, *VEGF* and *PD-L1* were shown. The Cp values were normalized to that of *GAPDH*, which was set to 100. *** *p* < 0.001 (ANOVA test) and # *p* < 0.001 vs. control.

We observed UA’s ability to inhibit hallmarks of cancer as well as tumorsphere formation. Here, we investigated the molecular signaling involved in these mechanisms. First, we examined whether UA could bind to the EGF receptor. Molecular docking analysis suggested an interaction between UA and EGFR with a strong binding affinity of −8.6 kcal/mol, suggesting that UA acted through EGFR signaling (Figure 5A). We tested this possibility by treating A549 and H460 cells with recombinant human EGF for 1 h, then treated the cells with or without UA, and analyzed the cell lysates using western blotting. We found an increase in the level of phosphorylated EGFR in the EGF-treated cells; such an increase was significantly reduced by 20 µM UA (Figure 5B). The level of EGFR remained the same for the control, UA-treated, and EGF-treated cells, suggesting that UA was bound to the EGF receptor and blocked its activation. Next, we analyzed UA’s effect on the JAK2/STAT3 pathway, one of the critical downstream targets of EGFR signaling. We found a suppression in the levels of phosphorylated EGFR, JAK2, and STAT3 by UA treatment in NSCLC cells. At the same time, the amount of total protein remained unchanged with increasing UA concentrations (Figure 5C). PD-L1 is also considered a downstream target for STAT3, which acts as a transcription factor for PD-L1. The inhibition of the expression of *POU5F1* by UA treatment also indicated that UA might act by regulating EGFR/JAK2/STAT3/PD-L1 signaling. Our previous results showed that UA could inhibit tumor angiogenesis, migration, and invasion (Figure 3). MMPs and VEGF play a vital role in tumor invasion and angiogenesis, respectively. As such, we analyzed the levels of MMP2, MMP3, MMP9, and VEGF in the NSCLC cells treated with UA and found a decreasing pattern for all these proteins (Figure 5D). We confirmed these results by analyzing the mRNA of these genes using real-time PCR, and found all these genes were similarly inhibited, much like PD-L1 *(CD274)* (Figure 5E). These results have delineated the molecular mechanism underlying the anticancer activities of UA.

### 3.6. UA Downregulates the Binding of STAT3 to MMP2 and PD-L1 Promoters

**Figure 6:**
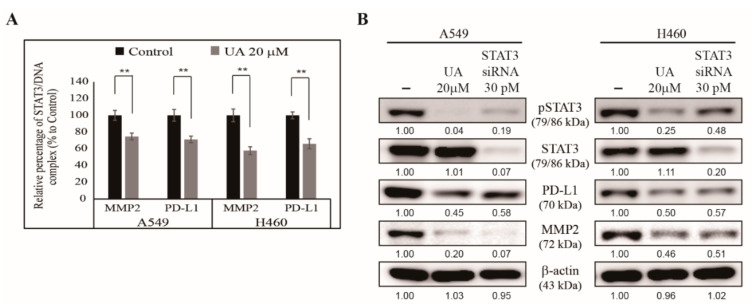
UA inhibited the binding of STAT3 to the MMP2 and PD-L1 promoters. (**A**) ChIP assay showed that treatment with 20 µM UA inhibited the formation of the STAT3/MMP2 and STAT3/PD-L1 complexes in A549 and H460 cells. The relative binding of STAT3 to the MMP2 and PD-L1 promoters was expressed as a percentage of the control. Statistical analysis was performed using Student’s *t*-test (** *p* < 0.01). (**B**) Western blotting of A549 and H460 cells treated with 30 pM STAT3 siRNA or 20 µM UA for 24 h showing the patterns of phosphorylated STAT3, STAT3, PD-L1, and MMP2 protein levels. β-actin was used as a housekeeping protein.

Previously, we showed that UA acted through the EGFR/JAK2/STAT3 signaling pathway through MMP2 or PD-L1 (Figure 5). Here, we hypothesized that the activated STAT3 was translocated to the nucleus and bound to the *MMP2* promoter to stimulate tumor cell invasion, and was bound to the PD-L1 promoter to boost tumor metastasis or regulate immune escape. We extracted chromatin DNA from the NSCLC cells with or without UA treatment, and analyzed the binding of STAT3 to the MMP2 and PD-L1 promoters. We found that UA significantly inhibited the binding of STAT3 to both promoters in the NSCLC cells (Figure 6A). These results suggest the vital role of STAT3 in the anticancer activity of UA. We tested STAT3’s role in UA’s activity by comparing the effect of *STAT3* silencing with that of UA treatment. We found a similar inhibition of phosphor STAT3, PD-L1, and MMP2 proteins in the cells treated with UA and the cells treated with STAT3 siRNA to that exhibited by the untreated control cells (Figure 6B). These results confirm that UA’s mechanism underlying its anticancer activity depends on STAT3 signaling, and that STAT3 acts as a bridge molecule between EGFR signaling and PD-L1 expression. 

**Figure 7:**
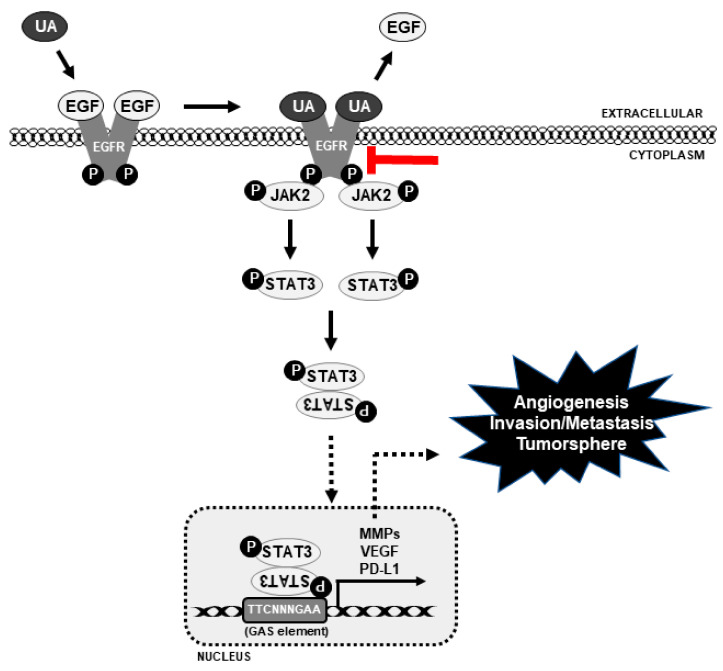
The molecular regulatory mechanism underlying UA’s anticancer activity in NSCLC cells, and UA’s inhibition of the EGFR/JAK2/STAT3 signaling pathway and PD-L1. Continuous black point arrow denotes the activity of UA, dotted point arrows denotes blockage of signals, and red block arrow denotes the inhibition of EGFR signaling by UA.

Altogether, the molecular mechanism underlying UA’s anticancer activity depends on EGFR signaling, which then signals to JAK2/STAT3, causing STAT3 to translocate to the nucleus to bind with the VEGF, MMP2, and PD-L1 promoters in order to block their transcription, thus inhibiting tumor angiogenesis, invasion, metastasis, and tumorsphere formation (Figure 7).

## 4. Discussion

Natural compounds have been used for cancer treatment for a long time. They could be used for targeted therapy and have the added advantage of causing no or fewer side effects [38,39]. UA is derived from several medicinal herbs, such as *Oldenlandia diffusa*, *Calluna vulgaris*, *Rosemarinus officinalis,* and *Eribotrya japonica* [40]. Many studies have demonstrated UA’s anticancer activities, such as inhibiting tumor angiogenesis and tumorigenesis, and promoting and inducing cell cycle arrest and apoptosis against various cancer cells, such as NSCLC, hepatocellular carcinoma, breast cancer, and gastric cancer [41,42,43]. UA also has an in vivo role; it has been reported to inhibit tumor growth in various animal models [44,45]. Although these studies suggest UA to be a drug candidate against NSCLC, the exact molecular mechanism of UA’s STAT3-dependent anticancer activity remains unclear.

The hallmarks of cancer mainly consist of the lost ability to induce apoptosis and the induction of angiogenesis, resulting in metastasis via tumor cell migration, invasion, and EMT. A drug that can inhibit these processes in a cancer cell will be suitable for anticancer studies [46]. We found that UA can induce cell death in A549 and H460 cells, which are both considered metastatic cancer cells. We observed that 20 and 30 µM UA induced almost 50% cell death in the A549 and H460 cells, respectively. From these, we chose 10 and 20 µM UA for the A549 and H460 cells, respectively, for further studies to demonstrate the concentration-dependent effects of UA. UA was also shown to alter the morphology of cells, inducing cell death. These results suggest UA as a drug candidate against NSCLC.

Uncontrolled cell cycle progression and escape from apoptosis leads to tumorigenesis. The ability to control cell cycle progression and apoptosis induction decides a cancer cell’s fate [47]. A natural compound that could induce cell cycle arrest and apoptosis against metastatic lung cancer cell lines is an appropriate candidate for anticancer activities. UA’s induction of G0/G1 arrest in A549 and H460 cells suggests its ability to act as an anticancer drug; cell cycle arrest leads to cell death. Inducing cell death with a natural compound is an effective therapeutic approach. Our results showed that UA also induced apoptosis in both NSCLC cells. Thus, these results provide strong evidence for UA’s anticancer activity.

When a tumor starts to grow, it may lack an adequate supply of oxygen due to its increasing size, thus creating hypoxic conditions through the hypoxia-inducible factor 1α, and then activating VEGF for angiogenesis. As a result of angiogenesis, oxygen will reach the tumor microenvironment through the blood supply [48]. Once the primary tumor forms, it starts to move to another location to create a secondary tumor. The tumor cells migrate and invade into blood vessels, and traverse through the bloodstream to spread the tumor. As such, tumor cell migration and invasion are considered as the initial steps in metastasis [49]. Our results showed that UA decreased in vitro angiogenesis by inhibiting tube formation in HUVECs. UA may disrupt tube formation, block vessel formation, reduce the oxygen supply to the tumor cells, and cause hypoxia. The analysis of UA’s molecular mechanism in HUVECs also showed UA’s inhibition of the VEGF and phospho-STAT3 expression. STAT3 plays a significant role in tumor angiogenesis by regulating VEGF activity [50]. UA was also shown to inhibit the migration and invasion of A549 and H292 cells, suggesting its role in suppressing tumor metastasis.

Anticancer drugs can induce apoptosis and inhibit angiogenesis and metastasis, while CSCs may remain inside silently. Upon stimulation, the CSCs differentiate into the primary tumor again. This condition is dangerous because the new tumor may be resistant to that particular drug [51]. Tumorsphere assays are efficient methods for studying CSCs and performing anticancer drug screening, as tumorspheres have the same characteristics as CSCs [52]. Here, we seeded A549 and H460 cells into DMEM/F12 media containing EGF, bFGF, and B27 in low-attachment plates with or without UA for the selective growth of tumorsphere. The bulk formation of the tumorsphere was observed in the control cells, indicating the presence of CSCs in the NSCLC cells. The presence of UA or STAT3 siRNA reduced the size of the tumorspheres, suggesting UA’s ability to inhibit CSCs via a STAT3-dependent signaling mechanism. SOX2, OCT4, and NANOG are considered the stem cell markers for determining tumorsphere formation [53]. The downregulation of the mRNA and protein levels of these genes in A549- and H460-derived tumorspheres by UA treatment indicated that UA could inhibit CSC markers. These results provide evidence supporting UA’s capability to target cancer cells as well as CSCs.

The knowledge of the molecular signaling mechanism underlying a natural compound’s activity is as essential as understanding its pharmaceutical effects. Molecular signaling begins with receptor binding, followed by the addition of the drug. EGFR is a crucial receptor in tumorigenesis, and mutations in EGFR result in tumorigenesis. It also acts as an intracellular tyrosine kinase domain essential for the signaling pathways that regulate cancer cell proliferation [54]. EGFR overexpression was observed in most NSCLC cells; regulating EGFR signaling could help control tumorigenesis or tumor progression. We hypothesized that UA could bind to EGFR, blocking its downstream signals. Molecular docking analysis suggested a strong binding between UA and EGFR. This prediction was confirmed by the observation that the level of phospho-EGFR was increased by treatment with human recombinant EGF, but was decreased by UA treatment. Thus, UA likely replaces EGF in its binding to the EGFR, thus blocking the activation of EGFR and abolishing downstream signaling through tyrosine kinases. JAK2/STAT3, a critical signaling pathway in tumorigenesis, is mediated through EGFR signaling; STAT3 can act as a transcription factor for tumor progression [55]. Consistent with our prediction of EGFR’s binding to UA, a reduction in the levels of phospho-JAK2 and STAT3 caused by UA was observed, whereas the total amount of JAK2 and STAT3 remained unchanged. These results demonstrate that the EGFR/JAK2/STAT3 signaling pathway is responsible for the anticancer activity of UA, and STAT3 plays a crucial role in the UA-dependent transcription processes.

*STAT3* is an oncogene that participates in most of the tumor development phases as a transcription factor. It is involved in tumor angiogenesis by regulating VEGF [56] and in tumor invasion by mediating MMP proteins [57]. The downregulation of activated STAT3 by UA was observed in NSCLC cells, suggesting that UA might block *VEGF* mRNA expression as it inhibited angiogenesis. Furthermore, UA might block *MMP* mRNA expression, as it inhibited tumor migration and invasion. As predicted, UA significantly downregulated the expression of *VEGF*, *MMP2*, *MMP3*, and *MMP9* mRNA, confirming its anticancer ability. *CD274*, encoding the immune checkpoint ligand PD-L1, is often overexpressed in NSCLC cells. Studies have shown that PD-L1 actively participates in tumor metastasis processes other than immune escape regulation [28]. Because of STAT3’s role in tumor metastasis, we hypothesized that STAT3 might also act as a transcription factor for PD-L1. Our results demonstrated the concentration-dependent inhibition of PD-L1 levels in UA-treated A549 and H460 cells, suggesting a possible relationship between STAT3 and PD-L1. As such, we analyzed the DNA-binding activity of STAT3 to the MMP2 promoter for tumor invasion, and the PD-L1 promoter for tumor metastasis.

We observed a significant reduction in STAT3/MMP2 and STAT3/PD-L1 complex formation in UA-treated NSCLC cells. Therefore, STAT3 plays an essential role in regulating PD-L1 in NSCLC cells. Moreover, UA exhibits anticancer activity by regulating EGFR/JAK2/STAT3 signaling and the expression of MMP2 and PD-L1.

## 5. Conclusions

In this study, we have demonstrated UA’s anticancer activity against NSCLC cells A549 and H460. We have determined that UA induces G0/G1 cell cycle arrest and apoptosis, thus inhibiting tumor angiogenesis, migration, invasion, and metastasis. Furthermore, UA has been found to inhibit the expression levels of VEGF, MMP2, and PD-L1 by regulating the EGFR/JAK2/STAT3 pathway. STAT3 has been found to act as a critical regulator in the anticancer activity of UA. Altogether, UA can be a drug candidate for PD-L1-based targeted therapy for NSCLC.

## Data Availability

The data presented in this study are available on request from the corresponding author. The data are not publicly available due to privacy.

## Supplementary Materials

The following are available online at https://www.mdpi.com/2227-9059/9/3/297/s1, Table S1: q-PCR primer sequences and annealing temperature.
