# Supplementary material for: Antitumor Effects of Ursolic Acid through Mediating the Inhibition of STAT3/PD-L1 Signaling in Non-Small Cell Lung Cancer Cells
Source: Biomedicines. 2021 Mar 13;9(3):297. doi: 10.3390/biomedicines9030297 (PMC7998465; doi:10.3390/biomedicines9030297)
Supplement: Supplementary file 1 [file biomedicines-09-00297-s001.pdf]

Sl No	Gene	Annealing temperature (°C)	Sequence (5` - 3`)
1	*CCND1*	58	F: 5′-tgtttgcaagcaggactttg-3′<br>R: 5′-tcatcctggcaatgtgagaa-3′
2	*CCNE1*	58	F: 5′-atcctccaaagttgcaccag-3′<br>R: 5′-aggggacttaaacgccactt-3′
3	*CDK4*	58	F: 5′-cccgaagttcttctgcagtc-3′<br>R: 5′-ctggtcggcttcagagtttc-3′
4	*CDKN1A*	58	F: 5′-atgaaattcaccccctttcc-3′<br>R: 5′-aggtgaggggactccaaagt-3′
5	*CDKN1B*	58	F: 5′-ccggctaactctgaggacac-3′<br>R: 5′-ttgcaggtcgcttccttatt-3′
6	*MMP2*	58	F: 5′-tgatggcatcgctcagatcc-3′<br>R: 5′-ggcctcgtataccgcatcaa-3′
7	*MMP3*	58	F: 5′-cacagacctgactcggttcc-3′<br>R: 5′-aggttctggagggacaggtt-3′
8	*MMP9*	58	F: 5′-ggacaagctcttcggcttct-3′<br>R: 5′-tcgctggtacaggtcgagta-3′
9	*VEGF*	58	F: 5′-aggagggcagaatcatcacg-3′<br>R: 5′-caaggcccacagggattttct-3′
10	*PD-L1*	58	F: 5′-tgccaggcattgaatctaca-3′<br>R: 5′-ggcctatttcctcctcttgg-3′
10	*GAPDH*	58	F: 5′-cccactcctccacctttgac-3′<br>R: 5′-tcctcttgtgctcttgctgg-3′
11	*MMP2* (ChIP assay)	58	F: 5′-tgcaggagcttgcttcttgt-3′<br>R: 5′-gcttgggtgcaaatccactg-3′
12	*PD-L1* (ChIP assay)	58	F: 5′-cccagctgcagcatctaagt-3′<br>R: 5′-aggccaaggtcaatgtgtct-3′

Table S1. q-PCR primer sequences and annealing temperature.
